# Rapamycin-induced metabolic defects are reversible in both lean and obese mice

**DOI:** 10.18632/aging.100688

**Published:** 2014-09-02

**Authors:** Yuhong Liu, Vivian Diaz, Elizabeth Fernandez, Randy Strong, Lan Ye, Joseph A. Baur, Dudley W. Lamming, Arlan Richardson, Adam B. Salmon

**Affiliations:** ^1^ The Sam and Ann Barshop Institute for Longevity and Aging Studies, The University of Texas Health Science Center at San Antonio, San Antonio TX 78245, USA; ^2^ The Geriatric Research Education and Clinical Center, South Texas Veterans Health Care System, San Antonio, TX 78229, USA; ^3^ Departments of Pharmacology, The University of Texas Health Science Center at San Antonio, San Antonio TX 78245, USA; ^4^ Institute for Diabetes, Obesity, and Metabolism and Department of Physiology, Perelman School of Medicine, University of Pennsylvania, Philadelphia PA 19104, USA; ^5^ Department of Medicine, University of Wisconsin School of Medicine and Public Health, Madison WI 53726, USA; ^6^ Reynolds Oklahoma Center on Aging, University of Oklahoma Health Sciences Center and Oklahoma City VA Medical Center, Oklahoma OK 73104, USA; ^7^ Departments of Molecular Medicine, The University of Texas Health Science Center at San Antonio, San Antonio TX 78245, USA

**Keywords:** rapamycin, glucose, insulin, obesity, mTOR

## Abstract

The inhibition of mTOR (mechanistic target of rapamycin) by the macrolide rapamycin has many beneficial effects in mice, including extension of lifespan and reduction or prevention of several age-related diseases. At the same time, chronic rapamycin treatment causes impairments in glucose metabolism including hyperglycemia, glucose intolerance and insulin resistance. It is unknown whether these metabolic effects of rapamycin are permanent or whether they can be alleviated. Here, we confirmed that rapamycin causes glucose intolerance and insulin resistance in both inbred and genetically heterogeneous mice fed either low fat or high fat diets, suggesting that these effects of rapamycin are independent of genetic background. Importantly, we also found that these effects were almost completely lost within a few weeks of cessation of treatment, showing that chronic rapamycin treatment does not induce permanent impairment of glucose metabolism. Somewhat surprisingly, chronic rapamycin also promoted increased accumulation of adipose tissue in high fat fed mice. However, this effect too was lost when rapamycin treatment was ended suggesting that this effect of rapamycin is also not permanent. The reversible nature of rapamycin's alterations of metabolic function suggests that these potentially detrimental side-effects might be managed through alternative dosing strategies or concurrent treatment options.

## INTRODUCTION

The mTOR (mechanistic target of rapamycin) signaling pathway serves as a central regulator of cell metabolism in response to nutrient and growth factor stimuli. The serine/threonine protein kinase mTOR acts as a catalytic core unit of both mTORC1 and mTORC2. Each mTORC complex likely plays discrete roles in metabolic function: mTORC1 integrates nutrient, growth factor and cellular energy status to regulate cell proliferation, growth and metabolism, whereas mTORC2 acts upon several downstream kinases including the Akt/PKB to regulate cell metabolism and survival [reviewed in [1]]. Due to its role in regulating cellular metabolism, aberrant mTOR signaling may be fundamental to the development of metabolic disease and dysfunction. For example, chronic activation of mTORC1 signaling in obesity is thought to play a significant role in the development of insulin resistance in muscle, adipose and liver tissue [2-4].

As the name implies, mTOR signaling is targeted by the bacterial macrolide rapamycin which interacts with the binding protein FKBP12 to inhibit some, but not all, mTOR functions [5]. While rapamycin is thought to primarily inhibit mTORC1 signaling through a direct mechanism, recent studies suggest chronic rapamycin treatment also down-regulates mTORC2 activity [6, 7]. Rapamycin and its analogues are approved for treatment of some forms of cancer and as immunosuppressants following organ transplantation. Rapamycin is also the first pharmacological agent capable of extending lifespan in both male and female mice according to the rigorous criteria established by the NIA's Intervention Testing Program [8-11]. Chronic rapamycin treatment has been shown to slow the progression of some, but not all, of the physiological declines associated with mouse aging [12-14]. Surprisingly, chronic rapamycin also promotes metabolic changes generally thought to be unfavorable, including glucose intolerance, insulin resistance and dyslipidemia, in several different rodent models [6, 15-20]. There is some evidence that the degree of metabolic dysfunction caused by rapamycin may be dependent on genetic background, length and means of administration of treatment and diet [6, 17, 18]. This is also a consideration in the clinical administration of rapamycin as the incidence of insulin resistance and new onset diabetes was shown to be significantly elevated in kidney transplant patients receiving rapamycin therapy [21, 22].

These potentially detrimental effects glucose regulation (glucose intolerance, insulin resistance, etc.) are concerns that currently may preclude the use of rapamycin and its analogues to treat and prevent age-related diseases. An important question heretofore unaddressed is whether chronic rapamycin treatment induces permanent alterations to metabolic function *in vivo*. In this study, we tested whether cessation of chronic rapamycin treatment could reverse its impairment of glucose metabolism in mice. In addition, we tested whether feeding mice a high fat diet, which also impairs glucose regulation, would exacerbate the impairment caused by rapamycin. Our results suggest that the metabolic effects of chronic rapamycin treatment are not permanent but rather dependent on its continued presence and activity suggesting that these adverse effects may be reduced or prevented through alternative treatment plans.

## RESULTS

### Chronic oral delivery of encapsulated rapamycin impairs glucose metabolism

**Figure 1:**
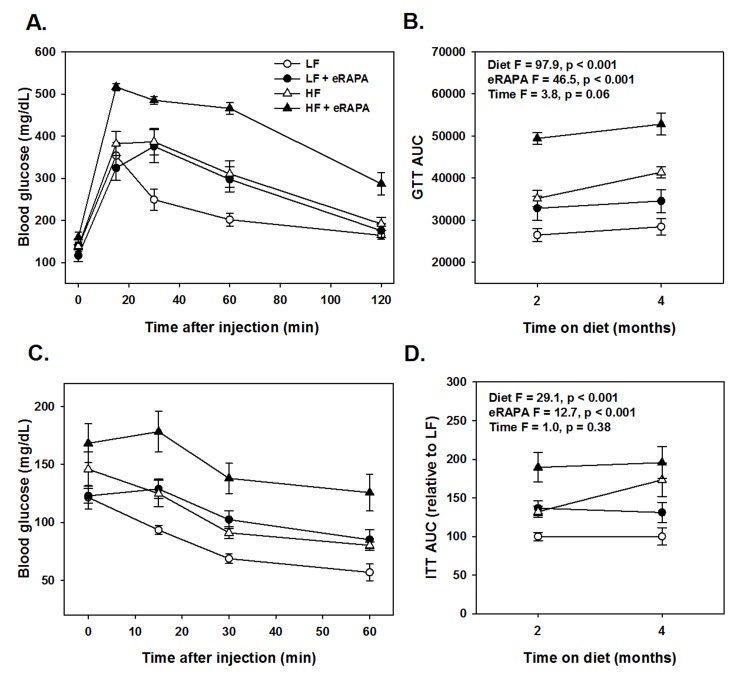
(**A**) Glucose tolerance tests for C57BL/6 males fed indicated diets for 2 months. (**B**) Area under curve (AUC) calculations for glucose tolerance tests following 2 or 4 months feeding indicated diets. (**C**) Insulin tolerance tests for C57BL/6 males fed indicated diets for 2 months. (**D**) AUC for insulin tolerance tests for insulin tolerance tests following 2 or 4 months feeding indicated diets. For all, symbols represent average (± SEM) values for n=6 mice at indicated time point for mice fed low fat (circle) or high fat (triangle) diets with (solid) or without (open) encapsulated rapamycin (eRAPA). F and p values are given for 3 way ANOVA testing indicated variables.

**Figure 2:**
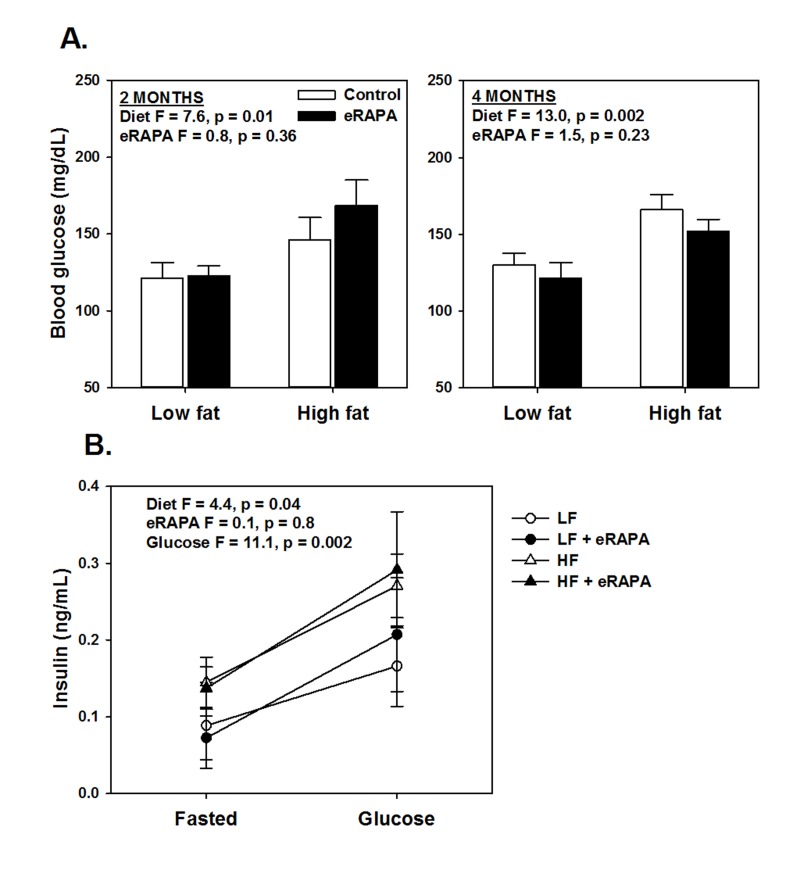
(**A**) Fasting blood glucose levels after feeding indicated diets for 2 and 4 months. (**B**) Fasting and glucose stimulated insulin levels in whole blood. For all, symbols represent average (± SEM) values for n=6 mice treated with (solid) or without (open) encapsulated rapamycin (eRAPA) for the indicated diet. F and p values are given for either 2 way (**A**) or 3 way (**B**) ANOVA testing indicated variables.

Chronic rapamycin treatment has been shown to impair several measurements of glucose metabolism including increased circulating levels of glucose and insulin and impaired glucose and insulin tolerance [6, 17, 18, 23, 24]. At least some of these effects seem to be dependent on genetic strain of the model system, means of administration and length of treatment [6, 17, 18, 23, 24]. Here, we found that chronic treatment with enteric rapamycin (eRAPA) significantly impairs glucose intolerance and promotes insulin resistance in C57BL/6 mice when given in combination with either a low fat or a high fat diet (Figure 1). The same group of mice were tested longitudinally, first after 2 months and then after 4 months of eRAPA treatment. Both high fat diet and eRAPA impaired glucose and insulin tolerance at each time points, but we found no significant interaction effect suggesting that eRAPA promoted metabolic dysfunction equally in both low fat and high fat diets (Figure 1B, D). Moreover, time on diet had no significant effect on either markers suggesting that the effects of eRAPA occur quickly but do not become progressively worse at least in the time frame at which we studied. Fasting blood glucose levels were unaffected by eRAPA at both time points on both diets, though high fat diet did significantly increase these levels (Figure 2A). We also measured glucose-stimulated insulin secretion to determine if this might explain the impaired glucose tolerance. While high fat diet significantly increased blood insulin levels in both fasted and glucose-stimulated mice, these levels were unaffected by treatment with eRAPA on both diets (Figure 2B).

**Figure 3:**
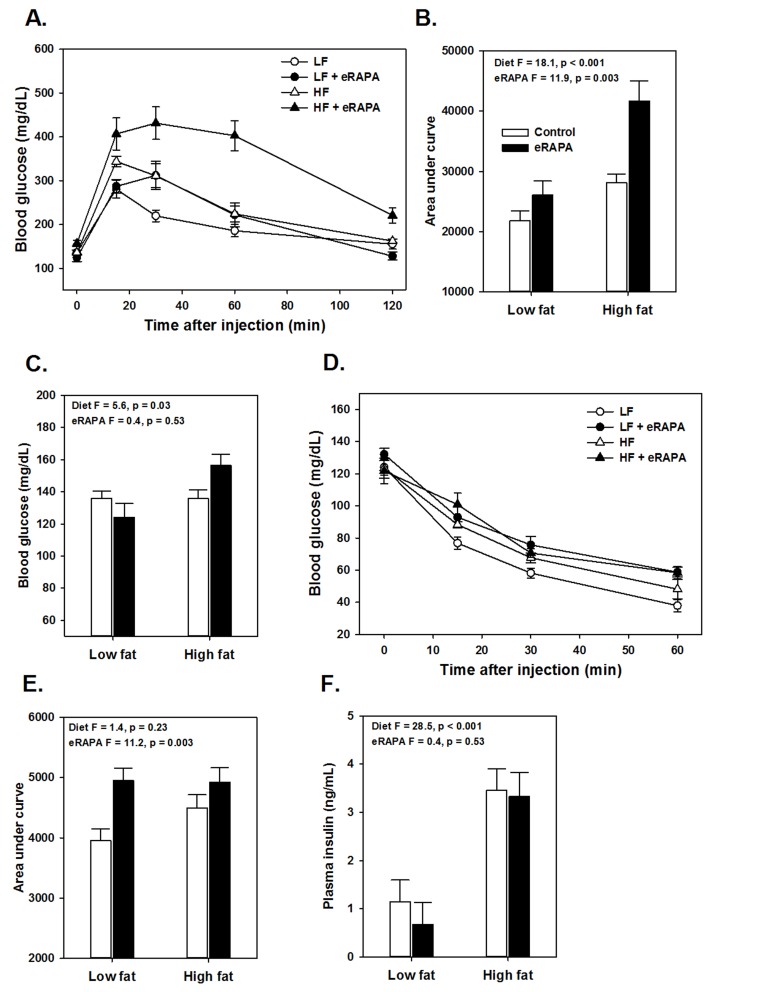
(**A**) Glucose tolerance tests for UT-HET3 males fed indicated diets for 3 months. (**B**) Area under curve (AUC) calculations for glucose tolerance tests shown in A. (**C**) Fasting blood glucose levels for mice in A. (**D**) Insulin tolerance tests for UT-HET3 males fed indicated diets for 3 months. (**E**) AUC for insulin tolerance tests for insulin tolerance tests. (**F**) Fasted plasma insulin levels. For all, symbols represent average (± SEM) values for n=6-10 mice at indicated time point for mice fed low fat (circle) or high fat (triangle) diets with (solid) or without (open) encapsulated rapamycin (eRAPA). F and p values are given for 2 way ANOVA testing indicated variables.

**Figure 4:**
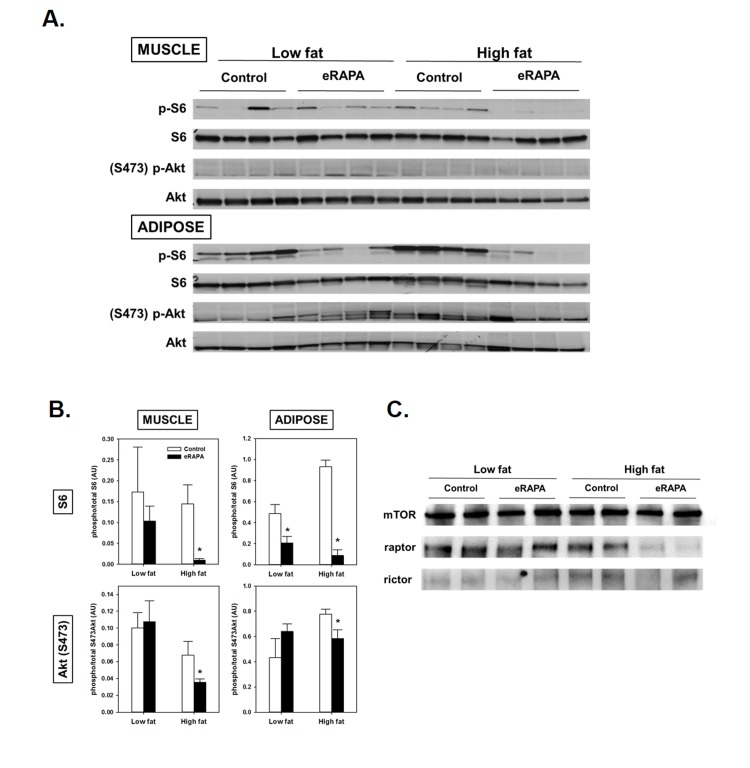
(**A**) Representative blots of p-S6, total S6, pAkt (Ser473) and total Akt in skeletal muscle and adipose of mice fed low fat or high fat diets with or without encapsulated rapamycin (eRAPA). (**B**) Quantification of relative phosphorylation of S6 or Akt (Ser473) of blots in A. Bars represent average (± SEM) values for n=4 mice treated with (solid) or without (open) eRAPA. Asterisks indicate significant difference between control and eRAPA group. (**C**) Representative blot of immunoprecipitation of mTOR from skeletal muscle protein homogenates and probed for the indicated proteins.

There is some evidence that the effects of rapamycin on insulin sensitivity differs among genetic strains of mice. For example, while inbred mice like C57BL/6 tend to become insulin resistant with rapamycin treatment, mice with heterogeneous genetic background have shown variable effects in terms of insulin sensitivity [6, 17, 18]. Here, we found that genetically heterogeneous UT-HET3 mice respond in a similar manner as C57BL/6 mice to chronic eRAPA in both diet formulations we used. In UT-HET3 mice, eRAPA impaired glucose tolerance and caused insulin resistance in combination with both low fat and high fat diets but did not affect fasting glucose or insulin levels (Figure 4). The degree of glucose intolerance caused by eRAPA in UT-HET3 mice was similar to that of C57BL/6 while insulin resistance was milder in the genetically hetero-geneous mice. Together, these data suggest the negative effects on glucose metabolism caused by eRAPA on glucose metabolism is largely independent of genetic background.

We found evidence that chronic eRAPA treatment significantly down-regulated both mTORC1 and mTORC2 signaling to a greater extent in high fat-fed mice compared to low fat fed mice (Figure 4). We assessed mTOR signaling in skeletal muscle and adipose tissue collected from UT-HET3 mice treated with eRAPA for 3 months. Both tissues showed significant reduction of phosphorylation of S6 in eRAPA - treated mice, indicative of inhibition of mTORC1 signaling. Chronic eRAPA treatment also inhibited mTORC2 signaling as measured by phosphorylation of Akt at Ser473; however, we only found this reduction of Akt phosphorylation in e-RAPA treated mice fed high fat diets. The interaction of mTOR with either raptor (mTORC1) or rictor (mTORC2) was reduced by eRAPA treatment with, again, a stronger inhibition in high fat-fed mice.

### Metabolism is normalized by ending rapamycin administration

**Figure 5:**
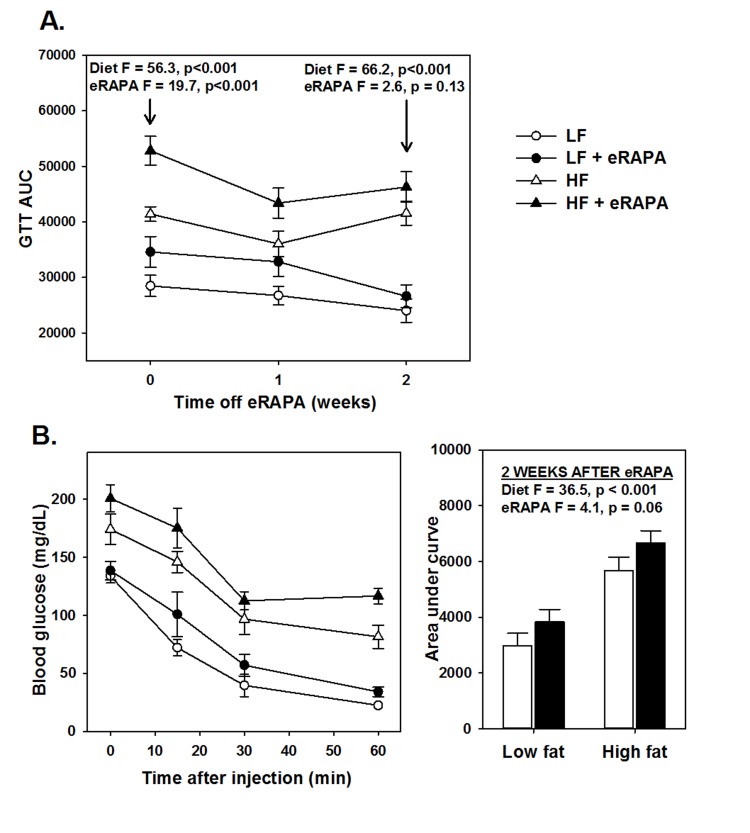
(**A**) Area under curve (AUC) calculations for glucose tolerance tests following 0, 1 and 2 weeks after cessation of encapsulated rapamycin (eRAPA) treatment. (**B**) Insulin tolerance tests (left) and AUC calculations (right) in mice 2 weeks after cessation of eRAPA treatment. For both, symbols represent average (± SEM) values for n=6 mice treated with (solid) or without (open) eRAPA for the indicated diet. F and p values are given for either 2 way ANOVA testing indicated variables.

An important question heretofore unaddressed is whether chronic eRAPA treatment induces permanent changes *in vivo* to physiological processes altered by rapamycin. We addressed this question by shifting C57BL/6 mice that had been treated with eRAPA for 4 months to their equivalent diets without eRAPA (i.e, mice fed high fat diet with eRAPA were now fed high fat diet without eRAPA, etc.). While treated with eRAPA, both diet (F = 56.3, p<0.001) and eRAPA (F =19.7, p<0.001) caused significant reduction in glucose tolerance in these mice (Figure 5A). Within 2 weeks of cessation of eRAPA treatment, glucose tolerance values were “normalized” to that of mice that had never been treated with eRAPA (Figure 5A). At this point, prior eRAPA treatment had no significant effect on glucose tolerance (F = 2.6, p = 0.13) while high fat diet still significantly impaired glucose tolerance (F = 66.2, p<0.001). Similarly, insulin sensitivity in these mice was relatively “normalized” within 2 weeks of cessation of eRAPA (Figure 5B). These data suggest that the impairment of glucose metabolism by eRAPA *in vivo* is reversible and can be mitigated by cessation of treatment.

### Chronic administration of encapsulated rapamycin promotes adiposity

**Figure 6:**
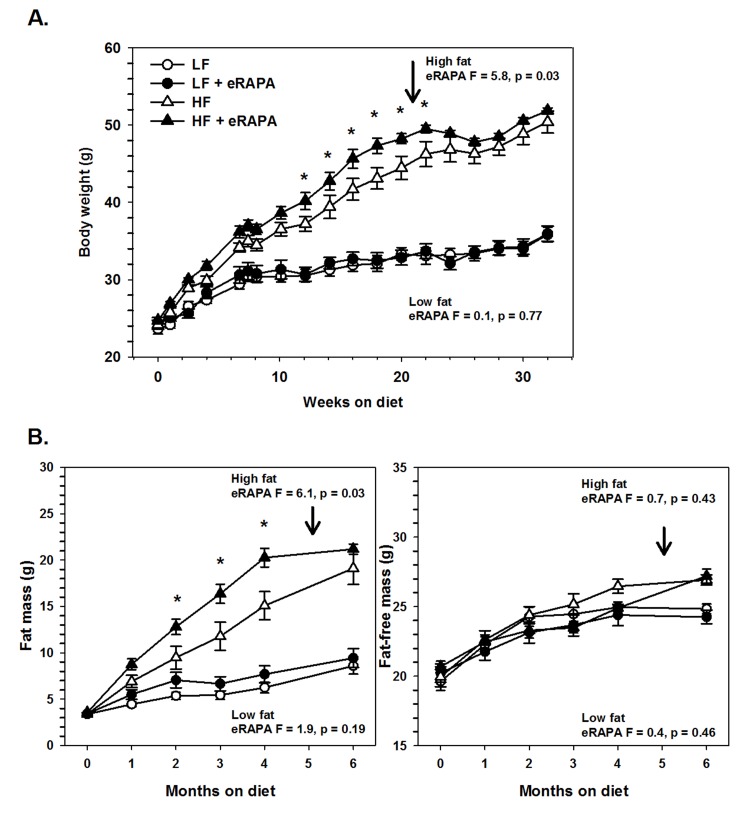
(**A**) Body weight of C57BL/6 males fed low fat (circles) or high fat (triangles) diets either with (solid) or without (open) encapsulated rapamycin (eRAPA). (**B**) Total fat content (left) and fat-free content (right) of mice in A. For all, symbols represent average (± SEM) values for n=6 mice. Downward arrow indicates time point of cessation of eRAPA treatment. F and p values given for repeated measures ANOVA testing the effect of eRAPA for the indicated diet. Asterisks indicate significant difference at time point between eRAPA and control for given diet from post-hoc analysis of ANOVA.

**Figure 7:**
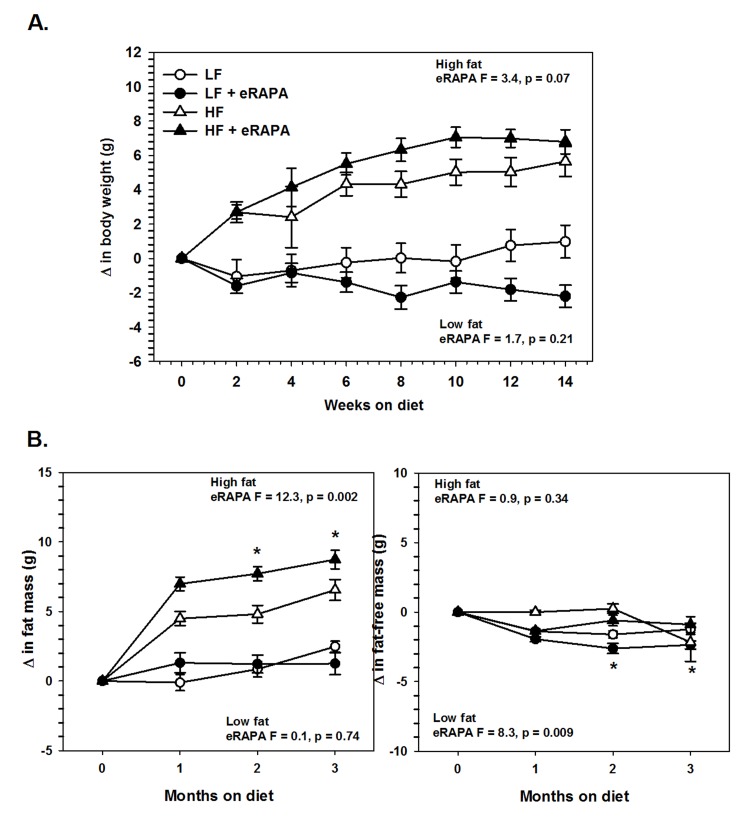
(**A**) Body weight of UT-HET3 males fed low fat (circles) or high fat (triangles) diets either with (solid) or without (open) encapsulated rapamycin (eRAPA). (**B**) Total fat content (left) and fat-free content (right) of mice in A. For all, symbols represent average (± SEM) values for n=6-10 mice. F and p values given for repeated measures ANOVA testing the effect of eRAPA for the indicated diet. Asterisks indicate significant difference at time point between eRAPA and control for given diet from post-hoc analysis of ANOVA.

Under our experimental paradigm, eRAPA when treated in combination with low fat diet had no effect on body mass, fat mass or fat-free mass (Figures 6 and 7). Contrary to reports suggesting that rapamycin treatment reduces body mass and adiposity [16, 19, 20, 25], we found that eRAPA treatment actually promoted increased adiposity in high fat fed mice. In high fat-fed C57BL/6 mice, both body mass and fat mass were significantly increased with continuous eRAPA treatment (Figure 6). Fat-free mass, likely consisting largely of muscle and bone, was unchanged with eRAPA treatment. We also found a similar increase in fat mass in high fat-fed UT-HET3 mice treated with eRAPA (Figure 7). Surprisingly, the differences in body mass and fat mass caused by eRAPA treatment were not permanent in C57BL/6 mice. Within only a few weeks of removing eRAPA from the diet, body weight and fat mass declined in high fat-fed mice previously treated with eRAPA such that there was no significant difference between this group and the high fat-fed “control” group of mice (Figure 6, arrow represents time of diet switch).

**Figure 8:**
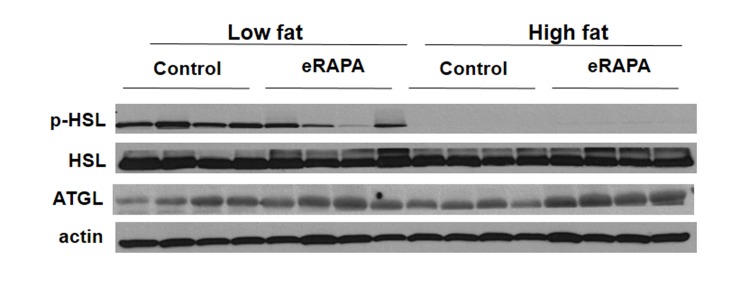
Representative western blot of p-HSL, HSL, and ATGL in adipose tissue from mice fed indicated diets

These data suggest that the obesogenic effect of eRAPA in combination with high fat or high caloric intake may be through pathways of fat mobilization. In vitro, inhibition of mTOR blocks adipogenesis and stimulates lipolysis [26-28]. In adipose tissue, we found that chronic eRAPA in combination with low fat diet reduces the phosphorylation of hormone sensitive lipase (HSL), the rate limiting step in lipolysis (Figure 8). In high fat-fed mice, there was almost no measurable phosphorylation of HSL in both control and eRAPA treated mice. These data suggest that lipolysis then is inhibited in vivo by chronic eRAPA treatment in mice fed the low fat fed mice. However, we also saw that adipose triglyceride lipase (ATGL), was elevated by eRAPA in both low fat and high fat diets suggesting increased breakdown of adipose resources (Figure 8). ATGL catalyzes the initial step in triacylglyceride hydrolysis, whereas HSL may have more specificity to the diacylglyceride form. These data then suggests a potentially complex effect of chronic eRAPA treatment that contributes to the adipose gain of these mice when also exposed to a high fat diet.

## DISCUSSION

One important side-effect of long-term rapamycin treatment in both rodents and humans is the potential for significant impairment of the normal regulation of glucose metabolism [6, 15-22]. Despite the potential of rapamycin both clinically and as an anti-aging therapeutic, the risk of new onset type 2 diabetes or other metabolic diseases is a significant obstacle for chronic use of rapamycin in humans. Our results confirm that chronic delivery of encapsulated rapamycin (eRAPA) does cause insulin resistance and glucose intolerance in both inbred and genetically heterogeneous strains of mice. Further, mice these effects are exacerbated in mice fed a high fat diet, supporting the notion that rapamycin treatment may drive the onset of type 2 diabetes. However, the key finding of this study is that the impairment of glucose metabolism by eRAPA in both low fat (lean) and high fat-fed (obese) mice are completely reversed by ending eRAPA treatment. This suggests that the administration of rapamycin through this paradigm does not induce permanent changes to the gluco-regulatory system. These data raise the possibility that metabolic defects caused by long-term rapamycin use could be mitigated transient withdrawal from the treatment, or by concurrent or alternative therapies. Festuccia et al. recently showed that rosiglitazone reduces hyperglycemia, glucose intolerance and insulin resistance caused by short-term treatment of rapamycin in rats [29]. It remains to be seen whether preventing metabolic dysfunction by treatments like this will further potentiate the beneficial effects of rapamycin in cancer treatment and prevention or longevity extension. There has been some suggestion that rapamycin-mediated modulation of different markers of glucose metabolism may differ depending on the time course of treatment, though this may be dependent on the model used and method of treatment. For example, Ye et al. showed that rapamycin treatment of C2C12 muscle cell lines had a biphasic effect on insulin response in that short-term treatment with rapamycin caused insulin sensitivity whereas long-term treatment caused insulin resistance [30]. This insulin resistance was associated with rapamycin-mediated inhibition of mTORC2 signaling. Similar to our work here, Lamming et al. also showed that a chronic, 2 week treatment of rapamycin in C57BL/6 mice caused glucose intolerance and insulin resistance that was, at least in part, mediated by down-regulation of mTORC2 signaling [6]. Houde et al. also found that 15 days of rapamycin treatment caused similar metabolic impairments in Sprague-Dawley rats [19]. Likewise, data from Fang et al. suggest that chronic treatment with rapamycin in a mixed genetic background results in sustained glucose intolerance, but found that insulin sensitivity as assessed by an insulin tolerance test was dependent upon the length of treatment, with mice treated for 20 weeks actually showing increased insulin sensitivity (albeit glucose intolerance) relative to control mice [18]. In contrast, Lamming et al. found that both short (3 week) and long (3 month) term treatment of female UM-HET3 mice with diet-delivered eRAPA caused glucose intolerance but neither treatment, nor treatment for over a year resulted in dramatic changes in insulin sensitivity [17]. It is a challenge to integrate the results from all of these studies due to difference in the genetic backgrounds of the models used, diets used and whether rapamycin was administered by injection or in an encapsulated form in the diet. Our data show no evidence of any metabolic switch with long term oral eRAPA treatment (similar to Lamming et al. [17]) suggesting that this may not occur with chronic oral delivery of encapsulated rapamycin. Also similar to Lamming et al., we found that eRAPA did not dramatically affect insulin levels suggesting that encapsulated rapamycin may not impair pancreatic function at least in the time-frame of these studies [18, 31, 32]. It remains to be determined whether this means of administration is essential for the reversibility of rapamycin's effects on glucose metabolism.

We found it surprising that chronic eRAPA treatment tended to promote increased adiposity in both high fat fed C57BL/6 and genetically heterogeneous UT-HET3 mice. Chronic activation of mTORC1 by obesity and metabolic stress appears to play a primary role in the development of insulin resistance and type 2 diabetes [3, 4, 33]. Inhibition of mTOR by rapamycin impairs adipocyte differentiation in cell culture [34]. Reduced mTORC1 signaling either in adipose tissue (by adipose-specific Raptor deletion) or through genetic ablation of the mTORC1 effector S6K1 prevents high fat diet-induced metabolic dysfunction [3, 4, 35]. Furthermore, some, though not all, studies have suggested that rapamycin treatment may be sufficient to reduce adiposity both in high fat fed, obese animals and in normal fed, lean animals [8, 16, 20, 23, 25, 36, 37]. On the other hand, inhibition of mTORC2 has been shown to negatively affect glucose regulation; deletion of Rictor in adipose tissue leads to weight gain and insulin resistance [38, 39], deletion of Rictor in liver leads to impaired glucose tolerance and increased gluco-neogenesis [6], and ubiquitous deletion of Rictor leads to hyperglycemia and hepatic insulin resistance [6]. Our data do suggest that rapamycin treatment is associated with reduced mTORC2 in high fat-, but not low fat-, fed mice suggesting a plausible mechanism for the increased adiposity in this group of mice. It seems also likely too that the development of insulin resistance in adipose tissue could contribute to the expansion of this tissue. It is also interesting to note that removal of eRAPA, and presumably the concurrent return of mTORC2 signaling and relative insulin sensitivity, completely reverses this phenotype.

The beneficial effects of rapamycin on lifespan, cancer and other diseases have largely been performed in models utilizing standard dietary conditions, i.e., normal, low fat rodent chow consisting of largely vegetable matter. In general, our results suggest that the physiological and cellular effects of chronic rapamycin are relatively similar even on diets containing differently levels of fat, and in fact may be actually exacerbated in conjunction with high fat diet. While more formal diet studies will be necessary, it seems likely that rapamycin may be effective under a variety of different conditions. In support of this, a recent study showed that the lifespan of C57BL/6 mice can be extended by rapamycin even when mice are fed a high fat diet [37]. However, the gluco-regulatory dysfunction experienced by mice treated with rapamycin while on a high fat diet highlights a potential concern with regard to the therapeutic use of rapamycin in humans, in which the dietary intake is much more varied in both content and composition.

The reversible nature of rapamycin's effects in this study also raises an important question about whether chronic treatment is required to reap the beneficial effects of rapamycin on longevity, cancer, etc.. It has been shown that rapamycin treatment extends longevity in mice to a similar degree whether it is started relatively early or late in life [8, 9]. Similarly, rapamycin impairs glucose metabolism in both young and old mice [17]. While these studies suggest that this compound may work equally well across ages, it is still not clear if short-term rapamycin treatments within a particular window(s) of time may cause persistent effects later in life. There is evidence that some physiological effects of dietary restriction, the most well-studied method to extend lifespan, are retained after this treatment is ended. For example, dietary restricted mice that are subsequently switched to an ad libitum diet retain significantly improved markers of glucose metabolism for months after this dietary modulation [40, 41]. In contrast though, the switch from dietary restricted to ad libitum feeding has also been shown to rapidly increase oxidative damage and alter the transcriptome to that of mice fed ad libitum their entire life [42, 43].

It has been suggested that the negative side effects of rapamycin treatment, including impaired glucose metabolism, will limit the use of rapamycin for the treatment of age-related diseases [44]. To our knowledge, the work presented here is the first to show that the metabolic defects caused by rapamycin are reversible after ending the treatment and suggests the possibility that the side effects of rapamycin could be minimized by short-term treatment with rapamycin. There is evidence that intermittent, rather than chronic, treatment with rapamycin is sufficient to extend lifespan in some mouse models [37, 45, 46]. Moreover, short-term or even single treatments with rapamycin have been shown to delay incidence or reduce prevalence in different mouse models of disease [46-48]. It will be of interest in the future to determine whether similar short-term treatments with rapamycin, or even treatment only at a few given points of life is also sufficient to extend lifespan and reduce disease burden in normally healthy mice without also causing potentially detrimental effects such as metabolic dysfunction.

## METHODS

### Animals

Male C57BL/6J mice were purchased from Jackson Labs (Bar Harbor ME) at 2 months of age and were randomly assigned to cages in our animal facility at a density of 3 mice/cage. Genetically heterogeneous UT-HET3 mice were generated at UTHSCSA using a cross previously described [8, 9]. Male UT-HET3 mice were used at approximately 10-12 months of age and housed at a density of 3-4 mice/cage. For both groups of mice, cages were randomly assigned to one of four different defined diets based on commercially available formulations. Both low fat (10% kCal from fat, D12450B, Purina/Test Diet, St. Louis MO) and high fat diets (45% kCal from fat, D12451, Purina/Test Diet) were prepared containing either encapsulated (enteric-released) rapamycin (eRAPA) or the eudragit vehicle (control) at concentrations of 14 ppm (mg of drug per kg of diet). eRAPA was provided through the San Antonio Nathan Shock Center of Excellence in the Basic Biology of Aging and details on preparation of eRAPA have previously been described in detail [8, 9]. Diets were provided *ad libitum*, mice were checked daily and food consumption and body weight were monitored bi-weekly. Body composition of non-anesthetized mice was analyzed by Quantitative Magnetic Resonance imaging (QMRi) using an EchoMRI 3-in-1 composition analyzer (Echo Medical Systems, Houston TX). For diet shift experiments, mice fed eRAPA-containing diet were given eudragit-containing control diet of the equivalent dietary fat concentration at the indicated time point.

### Glucose metabolism

Glucose and insulin tolerance tests were performed 2 and 4 months (C57BL/6) or 3 months (UT-HET3) after beginning dietary treatment. For glucose tolerance tests, mice were fasted 6 hours (09:00-15:00) prior to each test and then injected intraperitoneally (IP) with glucose (1.5 g kg^−1^) in saline. For insulin tolerance tests, mice were fasted 6 hours (09:00-15:00) prior to each test and then injected IP with insulin (1 U kg^−1^) in saline. Blood glucose levels were measured at indicated time points from tail vein bleeding by hand-held glucometer (LifeScan, Milpitas CA). Area under curve (AUC) was calculated for each animal using the Trapezoid method. For glucose-stimulated insulin secretion, mice were fasted 6 hours (09:00-15:00) prior to each test and then injected intraperitoneally with glucose (1.5 g kg^−1^) in saline. Whole blood was collected in EDTA-containing tubes from the tail vein prior to and 15 minutes after injection with glucose as previously described [6, 17]. Insulin levels in whole blood and plasma were measured using Crystal Chem ultra-sensitive mouse insulin ELISA (Downer's Grove IL).

### Immunoblots

Total protein extracts were isolated from skeletal muscle (gastrocnemius) and visceral adipose (epigonadal) that was collected from mice, snap-frozen in liquid nitrogen, and stored at -80º C until use. Mice were fasted overnight and sacrificed 10 minutes after IP injection of insulin (1 U kg^−1^). Protein extracts were made in RIPA buffer with added protease and phosphatase inhibitors (Thermo Scientific, Rockford IL), centrifuged at 13,000 g and 4º C for 15 min, then stored at -80ºC until use. Total protein content was measured by the Pierce BCA assay (Bio-Rad, Hercules CA). Proteins were separated by SDS-PAGE and transferred to PVDF membrane for immunoblotting. Phospho-S6, S6, Phospho-Akt (ser473), Akt, mTOR, raptor, rictor, phospho-HSL, HSL and ATGL antibodies were from Cell Signaling (Beverly MA). Actin antibody was from Sigma (St. Louis MO). For immuno-precipitation, muscle samples were lysed in cold 0.3% CHAPS lysis buffer [40 mM Hepes (pH 7.5), 120 mM NaCl, 1 mM EDTA, 0.3% CHAPS, 10 mM pyrophosphate, 10 mM β-glycerophosphate, 50 mM NaF, 0.5 mM orthovanadate, and protease inhibitors], then centrifuged 16,000 rpm for 15 min at 4^°^C. Protein A agarose beads were added to the supernatant and incubated with rotation for 1 h, centrifuged and mTOR antibodies were added to the cleared lysates. After overnight rotation at 4^°^C, protein A agarose beads were added incubated at 4^°^C for an additional hour. Immunoprecipitated complexes were washed in 0.3% CHAPS lysis buffer three times, boiled in SDS-sample buffer, separated by SDS-PAGE, and analyzed by immunoblotting. Protein bands were visualized by ECL and densitometry analyzed using Image J.

### Statistical analysis

For glucose tolerance, insulin tolerance, and plasma glucose and insulin measurements, the effect of both diet (low fat vs. high fat) and rapamycin (control vs. eRAPA) were analyzed using two way ANOVA. Longitudinal studies in C57BL/6 mice were analyzed by three way ANOVA to determine effect of diet, rapamycin and time (2 mo. treatment vs. 4 mo. treatment). Glucose stimulated insulin secretion was assessed using three way ANOVA to determine effect of diet, rapamycin and glucose injection. Body weight, fat mass and fat-free mass were analyzed by repeated measures two-way ANOVA. Post-hoc multiple comparison tests were performed using the Holm-Sidak method. Immunoblots were analyzed by t-test comparing the effect of rapamycin within each diet treatment group.
